# How do hospital professionals involved in a randomised controlled trial perceive the value of genotyping vs. PCR-ribotyping for control of hospital acquired *C. difficile* infections?

**DOI:** 10.1186/1471-2334-14-154

**Published:** 2014-03-21

**Authors:** Ala Szczepura, Susan Manzoor, Katherine Hardy, Nigel Stallard, Helen Parsons, Savita Gossain, Peter M Hawkey

**Affiliations:** 1 Warwick Medical School, University of Warwick, Coventry, UK; 2 Faculty of Health and Life Sciences, University of Coventry, Coventry, UK; 3 Research and Development, Heart of England NHS Foundation Trust, Birmingham B9 5SS, UK; 4 School of Immunity and Infection, University of Birmingham, Birmingham, UK; 5 Public Health Laboratory Birmingham, Public Health England, Heart of England NHS Foundation Trust, Birmingham B9 5SS, UK

**Keywords:** Hospital infection control, *C. difficile* tests, Ribotyping, MLVA sub-typing, Value of test information, Staff attitudes

## Abstract

**Background:**

Despite scientific advances in typing of *C. difficile* strains very little is known about how hospital staff use typing results during periods of increased incidence (PIIs). This qualitative study, undertaken alongside a randomised controlled trial (RCT), explored this issue. The trial compared ribotyping versus more rapid genotyping (MLVA or multilocus variable repeat analysis) and found no significant difference in post 48 hour cases (*C difficile* transmissions).

**Methods:**

In-depth qualitative interviews with senior staff in 11/16 hospital trusts in the trial (5 MLVA and 6 Ribotyping). Semi-structured interviews were conducted at end of the trial period. Transcripts were content analysed using framework analysis supported by NVivo-8 software. Common sub-themes were extracted by two researchers independently. These were compared and organised into over-arching categories or ‘super-ordinate themes’.

**Results:**

The trial recorded that 45% of typing tests had some impact on infection control (IC) activities. Interviews indicated that tests had little impact on initial IC decisions. These were driven by hospital protocols and automatically triggered when a PII was identified. To influence decision-making, a laboratory turnaround time < 3 days (ideally 24 hours) was suggested; MLVA turnaround time was 5.3 days. Typing results were predominantly used to modify initiated IC activities such as ward cleaning, audits of practice or staff training; major decisions (e.g. ward closure) were unaffected. Organisational factors could limit utilisation of MLVA results. Results were twice as likely to be reported as ‘aiding management’ (indirect benefit) than impacting on IC activities (direct effect). Some interviewees considered test results provided reassurance about earlier IC decisions; others identified secondary benefits on organisational culture. An underlying benefit of improved discrimination provided by MLVA typing was the ability to explore epidemiology associated with CDI cases in a hospital more thoroughly.

**Conclusions:**

Ribotyping and MLVA are both valued by users. MLVA had little additional direct impact on initial infection control decisions. This would require reduced turnaround time. The major impact is adjustments to earlier IC measures and retrospective reassurance. For this, turnaround time is less important than discriminatory power. The potential remains for wider use of genotyping to examine transmission routes.

## Background

*Clostridium difficile* infection (CDI) is a major cause of hospital acquired infections (HAIs). Early detection and control of CDI is essential to reduce the likelihood of cross-infection and prevent serious outbreaks. The development of more rapid and discriminatory typing tests should improve infection control (IC). However, the evaluation of diagnostic tests is recognised to be complex, including technical, clinical and organisational dimensions [1,2]. The Diagnostic Assessment Programme recently established by the National Institute for Health and Care Excellence (NICE) also highlights the need for qualitative evidence on the experiences of staff who use new tests [3].

CDI occurs in patients in whom the normal gut flora have been destroyed, usually after use of a broad spectrum antibiotic. Patients undergoing general surgery [4], oncology patients [5,6] and those with chronic renal disease [7] are at particular risk of becoming infected during a hospital stay. *C. difficile* infection can be life-threatening, especially in elderly patients. Because of its long incubation period it is not always easy to determine with accuracy the origin of transmission or contamination [8-10]. On a hospital ward, the first indication of possible CDI transmission is a period of increased incidence (PII) or CDI cluster which, if not controlled, can turn into a serious outbreak. The United Kingdom (UK) Department of Health defines a PII as two or more new cases occurring on a ward (>48 hours post admission, but not relapses) in a 28-day period; an outbreak is defined as two or more cases caused by the same strain [11].

Determining whether transmission has occurred is essential for tracing of linked cases and effective infection control. This requires some means of typing strains to confirm whether cases are in fact linked. The discriminatory ability of a test is crucial in defining whether a second isolate is part of an outbreak or a sporadic infection. There are a variety of techniques that can be used to type *C. difficile* strains. PCR-ribotyping was the first method to gain widespread acceptance for this purpose [12]. In the UK, the Health Protection Agency (HPA) established a *C. difficile* Ribotyping Network in 2007 to provide a typing service for NHS hospital trusts. However, PCR-ribotyping may not provide enough discriminatory power to fully characterise *C. difficile* strains. Therefore, in 2008 the HPA introduced a new service to provide ‘enhanced fingerprinting’ of *C. difficile* for NHS hospitals [13]. Access to this service was strictly controlled, in the first instance by regional microbiologists, due to its cost and the need to balance availability with the scale of the challenge. The test used to provide this more detailed ‘fingerprint’ or genotyping information is multilocus variable repeat analysis (MLVA), sometimes termed multi locus variable number tandem-repeat (VNTR) analysis [14]. The MLVA test has a greater discriminatory ability than most other fingerprinting methods for analysing closely related *C. difficile* strains [15].

The epidemiology of *C. difficile* has changed with the emergence of new, virulent strains such as ribotype ‘027’ [16]. This has been associated with larger hospital outbreaks and more severe disease, resulting in a much higher mortality rate and increased risk of complications, as well as more recurrent infections or relapses. Typing of these more virulent strains is essential in order to correctly differentiate outbreaks with the potential for serious consequences from apparently related clusters. MLVA can distinguish more than 20 sub-types of ‘027’, now one of the more commonly occurring *C. difficile* ribotypes [17]. Importantly, the method can also provide a high level of discrimination among other important epidemic *C. difficile* ribotypes, including ribotypes ‘001’ and ‘106’. Together with ‘027’, these accounted for approximately 70% of *C. difficile* isolates ribotyped by the NHS in 2007-08 [18]. Since 2009, National Health Service (NHS) hospital trusts have had to report every CDI outbreak to their local primary care trust (PCT) or strategic health authority (SHA); they may incur a fine of up to £50,000 if they are found to have breached hygiene regulations [19]. UK hospitals are also expected to subject outbreaks to a resource-intensive, root cause analysis in order to highlight any factors which might be linked to transmission and to take appropriate preventative and precautionary steps [20].

Infection control (IC) measures to manage CDI clusters necessitate a complex range of decisions to address staff behavioural aspects as well as organisational factors [21]. It has been argued that, without more discriminatory information on strains, hospital staff may incorrectly assume they have a CDI outbreak and may institute unnecessary and expensive IC procedures. In contrast, if typing can confirm that transmission has taken place, this may facilitate more timely IC measures and reduce the number of further cases. At present, there is a paucity of research evidence to indicate the degree to which typing results actually influence hospital staff in their infection control decisions or what the added benefits of more discriminatory MLVA information are when dealing with clusters of cases.

The present qualitative study was undertaken alongside a randomised controlled trial (RCT) set up to measure the differential impact on hospital CDI rates of MLVA typing versus standard ribotyping [22]. The trial demonstrated no significant difference between MLVA and control group in terms of a reduction the number of post 48 hour cases of *C difficile*. The qualitative study collected in-depth qualitative data from senior hospital staff on the use of genotyping and ribotyping information for control of hospital acquired infections during periods of increased CDI incidence.

## Methods

A total of sixteen hospitals were recruited to the main trial. All PIIs were identified and isolates sent for typing by MLVA or PCR-ribotyping. A ‘test-impact’ data collection form was attached to each typing result returned (see Additional file 1). This short questionnaire requested information on whether, as a consequence of the typing information, any infection control actions were initiated, or whether any actions had been stopped. The questionnaire also asked respondents to categorise the degree to which each typing result had aided infection control management (strongly agree, agree, disagree, strongly disagree). Hospitals involved in the trial followed their own protocols for initial identification of *C. difficile* toxin-positive patients and subsequent infection control procedures for CDI [22].

A qualitative study was undertaken alongside the main trial in order to explore test utilisation in greater detail. All hospital trusts recruited to the RCT were invited to take part in the study. Lead microbiologists in each trust were sent an information sheet about the study and asked whether they, or another suitable member of the hospital’s infection control staff, would be prepared to be interviewed. Interview trusts were selected using maximum variation sampling to ensure that their characteristics were representative of the whole trial population [23]. The final sample comprised eleven trusts covering a total of 20 hospitals.

A topic guide (Table 1) was developed based on analysis of test-impact forms and piloted before use. Interviewees were sent the topic guide prior to interview together with a breakdown of their trust’s test-impact responses over the time period of the trial (April 2010-April 2011). The qualitative study was conducted at the end of the trial period (July-September 2011) in order not to influence trial outcomes. Participants were free to choose the date and time for their interview. All interviews were undertaken by the same person (a senior researcher with over twenty years’ experience of evaluating diagnostic tests). Interviews, which lasted 45 – 90 minutes, were audio-taped with the participants’ consent and later transcribed verbatim. Each participant was allocated a study number to ensure confidentiality. Additional notes on the interviews were taken by the interviewer.

**Table 1:** Interview topic guide

**Interview topics**	**Questions to ask**
Ribotyping history	When first offered a ribotyping service
	Benefits of ribotyping when dealing with periods of increased CDI incidence
	Weaknesses of a ribotyping service
MLVA typing service	Has/would access to more rapid MLVA information be of added benefit and, if so, how?
	*What factors, if any, have limited value of MLVA information? (MLVA Group only)*
Initiating ward closures	You reported XX ward closures as a result of ribotyping/MLVA *t*yping result
	Explain decision-making process for initiating a ward closure.
	How has/could ribotyping/MLVA typing influenced decision-making in this area
Initiating extra cleaning	You reported XX instances of extra cleaning as a result of ribotyping/MLVA typing
	Explain decision-making process for initiating cleaning changes.
	How has/could ribotyping/MLVA typing influenced decision-making in this area
Stopping ward closures/extra cleaning	Value (if any) of ribotyping/MLVA typing in preventing ward closures or changes in cleaning
	How ribotyping/MLVA typing influences decision-making
Changes to audits of practice/staff training	Please explain decision-making process in the areas of audits of practice and staff training.
	How has/could ribotyping/MLVA typing influenced decision-making in these areas
Other changes	Changes which could improve value of typing information further in infection control and why

Transcripts were analysed using framework analysis [23] supported by NVivo-8 software package [24]. Two researchers examined the material using a qualitative thematic approach (the senior researcher and an experienced qualitative researcher). Analysis involved comparing different interviewee’s accounts with one another, and coding responses into recurring themes capable of accommodating all the data [25]. Close analysis of the transcript data identified a number of sub-themes. These were then grouped under a number of over-arching categories or ‘super-ordinate themes’. In cases where the researchers failed to agree either the categorisation was discussed until agreement was reached or the coding frame was modified to accommodate the new data. At this stage of the analysis sub-themes might exhibit elements which meant they could be placed in more than one category, indicating the inter-relatedness of some themes. In these cases, one super-ordinate theme was identified and, if necessary, certain elements of the sub-theme and the associated quotations were assigned to a different sub-theme. Theoretical saturation was considered to have been reached once no new theme emerged [23].

The research study was carried out in compliance with the Helsinki Declaration (http://www.wma.net/en/30publications/10policies/b3/index.html). Ethical approval was received on 08/01/2010 (reference number 10/H1202/3) from the Black Country Research Ethics Committee which had UKCRN approval; R & D approval for all 16 trusts was also obtained.

## Results

### Characteristics of interview sample

Eleven hospital trusts agreed to participate in the qualitative study; five were in the intervention group and had experience of MLVA and PCR-ribotyping and six were control sites with experience of ribotyping only. Hospitals identified 15 staff who performed an infection control role in their trust for interview. These included eight directors of infection prevention/control, six consultant microbiologists and one nurse member of the hospital infection control team. Table 2 shows that the trusts in which individuals were located were representative of the whole trial population in terms of size (number of beds) and on-site access to hospital isolation facilities.

**Table 2:** Characteristics of trusts participating in interviews vs. all trial trusts

	**Interview group**		**Trial population**		**All trusts in trial**
	**MLVA**	**Ribotyping**	**MLVA**	**Ribotyping**	
Number Trusts Interviewed	5	6	NA	NA	NA
**Hospital isolation facilities**					
Average bed size	1,056	977	922	912	902
Single rooms with en-suite (*% total beds*)	10%	10%	14%	13%	12%
Single rooms without en-suite *(% total beds*)	10%	6%	10%	7%	5%
**Average typing test requests over 12 months**					
Test requests (*mean number/trust*)	56.0	61.5	66.8	58.6	50.4
**All post 48 hr *C difficile *** **cases reported over 12 months**			814	868	1,682

Table 3 provides an overview for the whole trial population in terms of the types of effects reported on trial test-impact forms. Overall, 16% of tests were reported to lead to additional cleaning measures, 12% to further audits of practice and 12% to additional staff training. The activity most often stopped was a planned audit of practice (20% of tests), followed by cleaning (10% of tests). Test-impact response patterns for trusts recruited to the qualitative study were similar to those reported by all the trusts in the trial.

**Table 3:** Direct impact of typing test results on infection control (IC) activities*

**Impact on infection control activity**	**Percentage typing results reporting effect (%)**	Type of effect reported** (% all typing results)			
		**Cleaning**	**Ward closure**	**Audit of practice**	**Staff training**
All IC activities	45	26	2	32	13
IC activity started	23	16	2	12	12
IC activity stopped	23	10	0	20	1

*Reported in 244 test-impact data collection forms attached to typing results over period April 2010 – April 2011 (120 ribotyping & 124 MLVA).

**More than one type of change may be recorded per test-impact form.

The two trial groups exhibited no difference in time from PII to receipt of samples in the typing laboratory (14.0 and 14.4 days for control and MLVA group respectively). However, laboratory turnaround time was significantly shorter for MLVA samples (5.3 days compared to 13.6 days for PCR-ribotyping; p < 0.001).

### Common themes emerging from interviews

An overview of the sub-themes drawn out from the transcript material is presented in Table 4. These were organised under four super-ordinate themes. Interviews focused on the factors which influenced infection control measures in their trusts and the added value of typing results when dealing with PIIS. In addition, intervention trusts were asked to consider their experiences of MLVA and compare this with the previous ribotyping service. In the PCR-ribotyping group, interviewees were asked what they considered might be the benefits of access to more rapid and discriminatory MLVA typing.

**Table 4:** Thematic categories & associated sub-themes from in-depth interviews

**Super-ordinate theme**	***Descriptor***	**Containing these sub-themes**
**1 Context**	*Internal trends and changes in external environment*	a) Fall in numbers of CDI cases
		b) Low transmission rates
		Requirement to report outbreaks
**2 Test process**	*Factors associated with the test itself which inhibit or facilitate utilisation of typing test results in the hospital*	a) Routine hospital protocols
		b) Timing of results
		c) Optimum lab turnaround time
		d) Process time in hospital
		e) More discriminatory typing information
		f) Understanding MLVA results
		g) Confidence in MLVA results
		Organisation of typing test requests
**3 Infection control**	*Interface between typing test result and specific infection control measures*	a) Ward cleaning
		b) Ward closure
		c) Audit of practice
		Staff training
**4 Indirect benefits**	*Effects not related directly to individual cases, including potential benefits*	a) Organisational culture
		b) Reassurance/confidence building
		Epidemiological value

#### Theme 1: context

In all interviews, the changing context was highlighted as an important factor influencing approaches to CDI control, with three main underlying sub-themes emerging.

##### Sub-theme: fall in numbers of CDI cases

Interviewees in both groups (MLVA and ribotyping) commented on a recent fall in UK numbers of CDI cases. This was perceived to have had both positive and negative impacts. It had made control of CDI more manageable in the trust while, at the same time, reducing the usefulness of typing tests because the hospital was no longer faced with such a large number of PIIs:

*It sort of falls into two time frames… One where we were clearly dealing with higher numbers than we are now, in which case it [typing] was really quite useful. I think the past 12 or 18 months it’s become much less so…because the incidence has fallen considerably* [4: Ribotyping Trust].

Set against this general trend of falling CDI numbers, an increase in faecal samples sent to the hospital laboratory might be triggered by local factors. Against small background number this could lead to an apparent rise in CDI incidence which was confusing. Factors mentioned in interviews included audits by infection control teams which led to apparent increases in the number of CDI cases in a hospital:

*[infection control nurses] remind them to send samples and then, you know, you get more samples, and you get more positives, and it’s almost a vicious circle. So I think there’s some sort of false clustering as well which makes things very, very confusing* [5: Ribotyping Trust].

At the same time, a decrease in overall CDI incidence did not necessarily lead to resource savings because the remaining cases might now be explored in greater detail:

*I think that’s what happened is that the numbers of cases of C. diff have dropped, so the amount of time we have put into each area, or each case, is substantially more* [27: MLVA Trust].

##### Sub-theme: Low transmission rates

Almost all interviewees pointed out that typing had identified that most PIIs did not involve linked cases, with 72% of PIIs in the trial (a similar percentage in both arms) shown not to be related. A growing expectation that this would be the case was considered to have influenced the value of typing information in decision-making:

*Well I think it’s been fascinating because our results have shown that clusters of similar types almost never occur… I’d have said that by chance we should have had more the same than we had – they are always different.…I mean I can’t give you the exact figure, but the number of times they’ve come back the same has been so small it’s almost as if, you know how Gregor Mendel fixed his result, I’d have said by chance we should have had more that were the same than we had. They are always different* [2: Ribotyping Trust].

*I think there was only one occasion where I think we had similar VNTR [MLVA] type on … one of our wards. Two cases, but apart from that most of the time anyway we have sent stuff over to you then it came back as different ones.* [17: MLVA Trust]

*no I suppose the only thing, pragmatically now is that the numbers we’re dealing with are now so low that it’s almost not of academic interest. I mean just looking back at this month we’ve had three hospital attributable cases of C. diff, three different wards, probably two of them well came in with it* [104: Ribotyping Trust].

##### Sub-theme: requirement to report outbreaks

Because more discriminatory MLVA tests identified fewer cases as linked, trusts perceived this as of direct financial benefit. This reduction in the number of outbreaks identified meant that fewer had to be reported to the PCT or SHA. In its turn, this reduced the trust’s exposure to possible fines:

*Obviously if they [PIIs] are outbreaks then PCT come into action and then we send the reports. So if the PII is not confirmed as an outbreak it’s a bit of a [financial] relief to us. … Especially when .. at the end of the year we calculate how many PIIs we declared how many were outbreaks. Last year we had 50% reduction in outbreaks - more than 50% reduction* [22: MLVA Trust].

Equally important, it could also reduce the hospital’s workload because effort was not wasted unnecessarily on non-serious incidents:

*…and if it’s called a definite PII then it becomes a serious incident, ‘serious untoward incident’. That gets reported to the SHA and the SHA would like to report all of these as serious but it’s a lot of work. We would like to know what the typing is first before, before you escalate. Because otherwise you give them a whole load of things but then you have to de-escalate and it’s an awful lot of work for our risk team* [66: Ribotyping Trust].

#### Theme 2: test process

Inevitably, infection control decisions are complex and do not depend solely on typing test information. Interviewees were asked to describe the degree to which typing results actually influenced their management of PIIs or CDI clusters. They were asked to consider any factors, including test characteristics, which had limited or enhanced this impact.

##### Sub-theme: routine hospital protocols

Interviewees considered that the initial decision of whether to *initiate* infection control measures could not be directly driven by typing results. Instead, both groups reported that once a PII was identified in their organisation, control measures were triggered by pre-existing hospital protocols. This was viewed as inevitable in a situation where test turnaround time could be longer than average patient length of stay:

*We’ve instituted a much more standard approach…..we use this horrible phrase ‘special measures’ so that we actually put heightened infection prevention input into a ward where we have two cases within a 28 day period irrespective of what the ribotyping ultimately reveals which is why …. the answer to the questionnaire has always been ‘no it hasn’t made any difference’* [12: Ribotyping Trust].

In some situations, control measures might even start with the first confirmed case, rather than waiting for a PII because ICD numbers were now so low. This could further limit the direct impact of typing:

*Whenever we test if there’s a positive on any of them we react exactly the same way. So we inform the ward, we inform the consultant, yep, we’ll do an RCA [root-cause analysis], and because we’re at the numbers now where we can you know do a meaningful RCA on each one….. we’ll do a full root-cause analysis* [37: MLVA Trust].

*And what we tend to do now is we have what we call a ‘slice of PII’ here. Which is you have a single case what we do is we go down at that stage and check the ward environment, then cleaning it triggers an automatic intervention to try and go and see if there are any things that we should be worried about at that stage. Because if we’ve got a case we want to make sure that our standards are high, although it might be too late. Then if we get two cases we automatically go and review everything and we do a terminal clean of the ward* [100: MLVA Trust].

*When we’ve got someone who’s got C. diff then they’re a very high priority to get a side room and that side room is cleaned daily with Actichlor Plus and then ideally we’ve got in our plan once a week the C. diff patients in a side room are moved to another side room, and the side room moved from gets cleaned with hydrogen peroxide.* [43: MLVA Trust].

##### Sub-theme: timing of results

All interviewees identified laboratory turnaround time as the main factor limiting the direct use and value of typing results. This was true for MLVA as well as for the slower ribotyping service:

*You’d usually make your decision and then when the ribotyping comes back, hopefully, it would confirm that you’d done the right thing* [11: Ribotyping Trust].

*If we get real time, I mean … if I could get the help quickly, urgently then probably it would really help for making those decisions really. … Because um even though this service was quick, it’s not quick enough to sort the things out* [48: MLVA Trust].

*In terms of how it actually helps us manage the outbreak, it doesn’t help us in real-time because we don’t get the results back in real-time* [6: Ribotyping Trust].

*I think the information obviously is not timely enough ..yes it’s a retrospective system* [7: Ribotyping Trust].

The time taken for typing the more rare ribotypes could prove to be quite lengthy:

*The other [thing] was the delay for some of the rarer ribotypes. So we actually had a couple of instances where um we were anxious, the people I think rather than me, were anxious to be reassured whether these were or not the same um and we couldn’t provide them with that information because of the delay* [97: Ribotyping Trust].

Interestingly, some interviewees reported that, over time, there was a growing confidence that CDI cases would prove not to be linked. This had reduced the perceived need for rapid typing information. Instead, results were used to provide reassurance:

*I suppose initially the main weakness was the time delay in getting the results back…..as this has gone on we have come to be, I don’t know, maybe falsely confident that they won’t be the same so it doesn’t really matter that it takes a bit of time* [8: Ribotyping Trust].

*But most of the time it turns out to be different VNTR [MLVA] types. So it was quite useful to reassure ourselves that it’s not a problem with you know … most might be sporadic cases really* [45: MLVA Trust].

Other interviewees remembered examples where a typing result had surprised them, but the turnaround time had limited its usefulness:

*…and to my surprise you know when we got the result back there did seem to be on-going transmission and that really focused management. But it would have been so much nicer to have been more timely* [57: Ribotyping Trust].

##### Sub-theme: optimum lab turnaround time

When asked to identify an ideal laboratory turnaround time, most interview subjects selected somewhere in the range 2-3 days. The average turnaround time recorded for the MLVA test (5.3 days) was nearly double the upper limit; and the 13.6 days recorded for ribotyping during the trial was almost five times higher:

*I don’t think it does need to be real-time. But … if we were able to get it sooner maybe we could change what our interventions were. So, perhaps if we know we’re going to get typing back in 2-3 days maybe there’s something in our standardised set that we might hold off on doing, or do differently. But again it’s speculation.* [90: Ribotyping Trust]

*You know, within 48 hours you can wait for a result. I think anything much longer than that you know you got a problem…* [60: Ribotyping Trust]

However, for some respondents even a 24 hour turnaround time might be too slow:

*At the moment I’d be really nervous around doing that [holding off action]. I think I’d be more comfortable around 24 hours. … I mean if we could have it in 24 hours then you’re gonna say okay you’re at risk here let’s just wait and see what [the result shows].* [21: MLVA Trust]

At the same time, hospital staff were clearly aware that such turnaround times might not be practicable with current technology:

*Well, in an ideal world…..you would want the turnaround time within a day. I know that’s absolutely not possible because you would have to grow things…* [10: Ribotyping Trust]

It was also acknowledged that expectations for a more rapid turn-around time with MLVA might in some cases not be met because of a necessarily drawn-out testing process:

*And sometimes one loci didn’t work, this didn’t work, and then you had to repeat it. So, it [time] was very much marginal difference between the two [MLVA and ribotyping]* [16: MLVA Trust].

##### Sub-theme: process time in hospital

Some respondents pointed out that a reduced turnaround time in the typing laboratory might still be of limited benefit. This was because of the extended time spent in the hospital prior to a sample being sent for typing. In the trial, an average period of two weeks was recorded from identification of a PII to receipt of samples in the central typing laboratory for both test groups. Some hospital staff were not always aware of the timelines:

*By the time the results come back it’s all ancient history. … when we’re requesting it, it’s quite retrospective already. They [patients] may be three or four weeks apart in time and we take a week to pick it [CDI] up. So by the time you get the typing results back the ward staff can’t even remember …* [9: Ribotyping Trust].

*It does sometimes seem to take quite a long time to get the results back. Some of this [overall time] might be due to being rather tardy in sending them off in the first place, I don’t know. I’ve got all that side of things taken care of by other members of staff so I don’t even have to initiate it now, and therefore I don’t necessarily know how timely it’s done* [52: Ribotyping Trust].

##### Sub-theme: more discriminatory typing information

A far higher percentage of MLVA typing results (41%) than ribotyping ones (10%) were rated ‘strongly agree’ in terms of the value recipients placed on their usefulness in aiding the management of a PII. It appears that, for MLVA, the more discriminatory information provided was of more value than any reduction in laboratory turnaround time:

*I’m very much impressed by the sub-type more than the rapid … .er the rapid perhaps didn’t help very much, really in terms of management* [35: MLVA Trust].

Respondents also explained that the value of MLVA typing information was greatest for more common ribotypes, where the previous test had been unable to discriminate, rather than for rarer ribotypes:

*I think 30-40% of our strains are 027 s. So if you get a few 027 s together, and you don’t know the sub-type, then you think well oh .. we know there’s a whole variety of things in there … then you’re much more worried about what’s actually going on. I think when you got a rare ribotype it’s less, yeah, it [MLVA] doesn’t add that much more of value….* [14: MLVA Trust].

*The biggest [advantage] would be things like the ‘020’ strain because that’s quite common for us to see quite a few people ‘020’ occasionally. Now sometimes it’s ‘005’ um so now we’ve got say three cases and we don’t know. It would be nice to go further and say are they definitely related* [65: Ribotyping Trust].

The enhanced ability to discriminate strains was also viewed as a distinct advantage when there was a larger outbreak or where an extended incubation period was in operation:

*The sub-typing was something which helps, especially if there’s a large outbreak..... like in our renal unit last year, we had five or six cases and it was getting quite difficult and I think at that point VNTR [MLVA] really helped to say, there were only two or three which were a linked sub-type* [18: MLVA Trust].

*So the incubation period can be quite prolonged… we’re worried that we might have problems up front and then people are dispersing all around …. Yes that typing was really useful, we learnt an awful lot from that and I’ve never seen something so clear cut as that* [20: MLVA Trust].

##### Sub-theme: understanding MLVA results

Although the regional laboratory provided an interpretation of MLVA results, local hospital staff still viewed the new test information as less straightforward. This meant that, unlike ribotyping, consideration of MLVA results often could not be delegated to more junior laboratory staff:

*It helps when she draws the [MLVA] trees and everything, and to actually say yes this is linked, to beautiful coloured pictures .. yes but for the common person in the lab and a quick result, and quick understanding the ribotyping is on your face, you just know it* [36: MLVA Trust].

*Yes I don’t think our laboratory staff should [be] involved in those results of VNTR [MLVA] so it’s just like a Consultant Microbiologist, a Senior Lab Manager, or those people, and I think it’s okay for us, yes….* [47: MLVA Trust].

*As it gets more complicated understanding the relatedness … it gets beyond me. Some of these other typing things. Is this a true difference or is it something that could have happened to the organism?* [31: MLVA Trust].

The ability to distinguish a recurrent CDI infection or relapse from a new infection was also important, although interviewees questioned whether even MLVA could distinguish this:

*And when we say it’s a relapse what is our confidence that it’s the same strain as they’ve had before. What’s to say they can’t have a different strain, I mean we call it a relapse but how do we know it’s a relapse of the same strain every time. I think it’s quite complicated actually, it’s not like dealing with, you know, MRSA outbreak on a ward* [33: MLVA Trust].

Often what was wanted was a simple ‘yes’ or ‘no’ answer:

*Because I mean now we’re here and we’re looking at people who are doing sequencing and clearly every germ is different from every other germ, so there’s a point at which you know you have to say well we don’t want to know you know there is stuff mutated … but something that tells us you know that it’s likely to represent transmission and that it’s unlikely …you know.* [61: Ribotyping Trust].

##### Sub-theme: confidence in MLVA results

In some instances, an MLVA test which indicated that cases were *not* linked might be questioned because of worries over sampling. This reduced confidence in the typing result and led to action based on the number of CDI cases:

*You know sometimes when you get the typing [MLVA] results back they’re all different but you suddenly have this massive, four or five cases on a ward and … you know… I suppose one of the worries is that when you’ve got somebody with C. diff is it actually you’ve just got a pure clone of one strain or is it actually because only one colony is picked off…. and I think so irrespective of the typing results if you get a blip and it’s beyond what you expect you go in there hard....* [15: MLVA Trust].

In other instances, if ribotyping and MLVA results disagreed, MLVA might not be believed and an outbreak recorded, especially for rarer ribotypes:

*I’m just trying to remember where ribotyping was same and VNTR [MLVA] was different and we didn’t call the outbreak… in fact the reverse has happened. This year VNTR called it different and ribotyping called it same and we still called it an outbreak, and that was ‘005’. Obviously the VNTR doesn’t have much experience with ‘005’ and we felt that this is quite unusual to have two on a ward … and then to have a slightly different VNTR … and we just didn’t feel confidence in their VNTR typing for that one. So we’ve felt that it looks clinically as .. as an outbreak and it fits in with the ribotyping, we will go ahead with the outbreak, and we called it as an outbreak, regardless of the VNTR* [36: MLVA Trust].

##### Sub-theme: organisation of typing test request

The process within a hospital trust for organising the typing test request was not necessarily straightforward. In some trusts, typing requests were managed entirely by a Consultant microbiologist. In these cases, interviewees appealed for a simpler and less time consuming request mechanism:

*Yes in my organisation, the Biomedical Scientists on the bench didn’t do the request for the typing. It actually went to a Consultant which is all very well. But I would find if I wanted to send three or four off it would probably take me near on three quarters of an hour, going in and out of each form. So, yes … the simpler the requesting mechanism the easier and perhaps if you are sending three or four could he just put it on one form or something* [62: Ribotyping Trust].

In one trust, the assumption that a senior Consultant would need to complete requests for MLVA tests was reported to have limited requests to the new typing service:

*I think that I was never really organised enough to, um we didn’t set up a system so I was talking to one of my colleagues in another hospital who has used it extensively. Unfortunately this was very recently and he said oh that’s fine, and all I had to do was tell the people in the lab send this one off, and this one off, and it was done. And I never put that in place, and I always kind of assumed that it would be totally my responsibility to do it all and therefore it never got done…* [40: MLVA Trust].

Even in cases where the consultant delegated the test request process itself to more junior staff, he or she might still have to support that member of staff in identifying appropriate cases:

*He [junior] will be reviewing the log sheet on a sort of you know two or three times a week basis and if he sees pairs coming up, which sometimes I also spot them myself because I also look at the sheets every week and I’ll send him an email saying have you spotted patients A and B or whatever, um so if he sees those occurring clusters of two on the same ward or connected to the same ward, within 28 days, he will then generate the request electronically and notify the laboratory that they are required to send off the isolates, so they get going digging out the specimens and sending them off* [53: Ribotyping Trust].

Because of the complex process required to identify CDI samples for typing, one interviewee suggested that it would be simpler if all *C. difficile* samples could be sent for typing:

*The other thing that I would think would be potentially beneficial would be just to expand the typing so that it was done routinely because it’s quite a lot of effort to try and work out which ones we should send and which ones we shouldn’t send* [95: Ribotyping Trust].

#### Theme 3: infection control

As well as exploring factors which influenced the general usefulness of typing test information, interviewees were also asked to explain the impact of results on specific IC measures in greater detail. In particular, they were encouraged to explain why typing results did, or did not, influence IC decisions during PIIs. Certain common themes emerged in interviews on the relationship between PCR-ribotyping and MLVA information and decisions about specific IC measures. These demonstrated the complexity of decision-making and primarily focused on implementing more targeted control measures over a period of time.

##### Sub-theme: ward cleaning

Interview groups explained that, rather than being driven by typing results, cleaning was routinely triggered by a PII. The process was pre-defined in some detail in all trusts, especially because contractors provide ward cleaning services:

*We have a fairly organised schedule of cleaning that happens now anyway. All the commodes get cleaned with a sporicidal agent routinely; all terminal cleans are with a sporicidal agent …… and I would say pretty much all C. diff cases have been isolated as soon as they’ve been diagnosed and then they’d have a terminal clean at the end of that period. So, we’re kind of doing the same thing regardless and the ribotyping didn’t really influence what we were doing in the end* [50: Ribotyping Trust].

*There’s a step change in the cleaning process so we have enhanced cleaning. Our cleaning contract is with [X: Company Name] and what we’ve negotiated with them is if there’s two or more cases on a ward of any infection then we have enhanced cleaning. That will mean um they will use a different product but they’d also [do] it more frequent during the day.* [72: MLVA Trust].

*We do that [enhanced cleaning] for any patient who has C. diff who is left in a single room with an en-suite now that low numbers of C. diff. It’s difficult in the winter when you’ve got flu, competing with it, and Norovirus. Yes, but we … use the Sterinis [H*_*2*_*O*_*2*_*] machine in the side rooms after they’ve vacated the rooms* [101: Ribotyping Trust].

Table 3 shows that additional cleaning was the most frequent impact reported. Interviews indicated that the cleaning action recorded was usually fine tuning of the pre-defined cleaning protocols. This most often involved ‘enhanced cleaning’: either increased frequency of cleaning or a switch of product e.g. to hydrogen peroxide (H_2_O_2_). In the smaller number of cases where typing results led to planned cleaning measures being stopped these usually involved not following through with a planned increase in frequency of cleaning or a change of cleaning product.

Although cleaning was largely governed by set protocols, more discriminatory information on CDI strains provided MLVA might trigger a closer subsequent look at a ward and this could lead to further actions, especially for wards with vulnerable populations:

*But I think where it [MLVA] also helps us … getting that [sub-type] was you know it’s still lurking there and you know you’re missing a trick somewhere. Going down there, lot of things came out, lot of things … because of the type of patients they were … because these are people who spend more time in hospital than they do at home. So they bring in everything bar the kitchen sink. So that prevents proper cleaning. They then go into a side room, all the hospital equipment gets clean, they then come out with all of their contaminated belongings. And we got rid of all of that* [28: MLVA Trust].

In cases where typing results confirmed an environmental problem, this could also be useful for negotiating extra cleaning hours or provide a lever for tackling long-standing estates issues:

*On one occasion, the discovery that two cases who had the same VNTR [MLVA] type had occupied the same bed on the ward served as an eye opener to the ward manager and other staff in relation to the importance of adequate cleaning* [44: MLVA Trust].

*And the other thing I was going to say is that the environmental audit might reveal some estates issues and if the ward’s on special measures we can use that as a lever to put pressure on the estates to resolve some of the issues that they might be loath to resolve otherwise* [98: Ribotyping Trust].

*Once you have the ribotype then you can quite confidently say that this is quite likely to be environmental, and we do want more cleaning hours in this ward* [64: Ribotyping Trust].

In one trusts, because existing cleanliness indicators used in the cleaning contract were shown to have failed to provide early warning of problems, a new scoring system was introduced:

*What we used to have, we’ve got [name of cleaning contractor] in here, but they’ve their own scoring system. If you look at the cleaning scores in this trust when they did it, all were between 97 and 99. […] So what was happening in this trust was that our early warning system, that there was a problem with the cleaning, was that we had a C. diff problem not vice versa. So, we have a vigorous system now where we performance manage and go around and look on the wards to make sure that they’re getting decent ICNA [Infection Control Nurses Association] scores* [30: MLVA Trust].

##### Sub-theme: ward closur

Ward closure represents a major cost to hospitals. Thus, if typing information can help avoid unnecessary closures this will provide significant financial benefits. As Table 3 shows, no-one indicated that a typing test had led to a planned closure being averted, but some interviewees did think this might be possible:

*So, if we have some evidence that these cases are not linked then probably we wouldn’t close the ward, or … I mean our actions will be slightly different. Not necessary everything will be different but this, this closing the ward is an issue* [103: MLVA Trust].

In reality, although more discriminatory MLVA information on CDI strains was potentially valuable, in one case a planned closure was not stopped because of the persistent nature of the problem:

*Somebody came to say to me, you know, why did you demand that ward was shut when they’re four different types. I can’t really say. But why do you do it when they’re all the same type and its on-going. Well, that’s bloody why, there’s something on that ward which is gonna do patients in. And we haven’t identified the force* [32: MLVA Trust].

Also, once set in motion, ward closure could be very difficult to stop. Contradictory typing information might not avert a planned closure. Especially, if this had been decided on clinical grounds:

*But for instance we, we recently had four cases of C. diff linked to our intensive care unit, which actually did lead to the ward being closed. […] The first time that had ever been closed in its history, and all the patients decanted. And we requested the VNTR [MLVA], and we managed to get the VNTR back really fast and they were all different. So actually yes […] we got 48 hour results back on that and very, very quick. But even that didn’t prevent the closure. Because the closure was done on clinical grounds with the number of cases.* [92: Ribotyping Trust].

Table 3 indicates that although typing results were reported to contribute to initiation of ward closures, this was relatively infrequent. An important factor emerging in interviews was a dramatic fall in CDI cases which reduced the need to close wards for this infection:

*I mean the first thing to say is the C. diff numbers in this trust have fallen like a stone. Now is very, very different from what we did then when we were right in the thick of it. It’s hard even to think back to that time because it’s so different now. Every ward had got it. If we’d closed - with all the C. diff - the entire hospital would have just closed down. We got to the point where we were setting up a separate ward for C. diff cases. But that was just at the point where the figures were on the turn. And we never had to do it* [49: Ribotyping Trust].

In interviews it became apparent that the definition of a ‘ward closure’ could range from complete closure and major refurbishment, especially if there was evidence of a persistent problem, to partial closure of consecutive bays. Typing information mainly contributed to decisions about partial ward closures:

*Um I don’t think we actually have ward closures as such. I mean, normally if we’ve had a PII then one of our first actions is to try to get hydrogen peroxide environmental decontamination on that ward… So although the ward might not have been closed technically, physically it has been closed because we’re trying to empty enough beds to be able to um to be able to clear a bay and then decant patients and then clear a bay.* [42: MLVA Trust].

*Sometimes people have closed bays and things but generally you don’t tend to do that unless you’ve got a huge problem. Now we did have a problem on one of the wards that was proved by ribotyping so it’s an increased incidence and that ended up with the entire ward being completely gutted and refurbished. And we haven’t had a problem since* [80: Ribotyping Trust].

Interviews also identified variations between trusts in the requirement to implement a full ward closure. Such closures were more likely in hospital settings with limited isolation facilities because this meant isolating patients decanted from individual bays was difficult:

*We only really did that once [closed ward] during the time the study was going on. And that was because we were in the old, it was actually in [Name of Hospital] where we had almost no isolation facilities and we had a suspicion that we had on-going spread that we weren’t able to control. We’ve never done one yet [since moving to new site] because the numbers that we’ve had are much smaller and … because we’ve got much better isolation facilities* [87: Ribotyping Trust].

Although staff were aware of the cost to the trust of ward closures, hospital capacity appeared to be a more important brake on closures:

*And that [ward closure] is an expensive business and very time consuming* [56: Ribotyping Trust].

*Closing wards is never an easy business, and as the wards are, we’re always being encouraged to have less and less beds, which is leading to huge problems. Yes, so you’ve got a higher occupancy rate which also has potential for increasing your C. diff rates, quite independently. But also its keeping wards closed when you haven’t got the capacity* [82: Ribotyping Trust].

One interviewee pointed out that even if the hospital policy required a ward to be closed and patients decanted, this might prove to be impossible due to the pressure on hospital beds:

*But our policy did say that in general if we thought that significant transmission had taken place, we would normally decant and use hydrogen peroxide vapour. But, it was always left up to management. Because there were times when [Hospital name] just you know was really busy and in the winter, and there were no wards you could decant to. So it wasn’t always an option* [59: Ribotyping Trust].

Once a decision had been made to fully close and deep clean an entire ward, the logistics and timing of implementing this decision could make the process very slow:

*We had to close the [Name] Ward and deep clean the entire ward and do the fogging, the hydrogen peroxide. That took more than a month to close before we were able to do that. […] If you want to clean and if you actually want to do hydrogen peroxide fogging you have to clean the entire ward; and to have thirty beds out of action the only time we could do this was a Christmas eve* [67: MLVA Trust].

##### Sub-theme: audit of practice

Table 3 shows that typing results were more likely to lead to a planned audit of practice being stopped, rather than a new audit started. As with ward cleaning, audits were usually set in motion as soon as a PII was declared. This was part of a battery of IC measures. Audits tended to be performed on a regular basis:

*So we always do regular audits of practice; the nurses go round and do those regularly. So we’ve got a very good infection control team here. So it wouldn’t be ribotyping really. We do go back in if there’s a PII. But that is again before the ribotyping comes back, and we look at practices because we can’t afford to wait then* [81: Ribotyping Trust].

*So the way we’ve been operating is that we have a weekly meeting where we review all of the previous week’s cases […] and we debate them and look at the timing, and so on and so forth, and declare whether it’s a PII or not, for that area. And then various things flow from that […] the auditing starts and the strain gets sent for ribotyping so really all of the, all of that sort of audit, extra cleaning and sending the strains for typing, that all happens at the same time. So, when the results come back, the results themselves haven’t really influenced very much.* [93: Ribotyping Trust].

Regardless of whether or not typing confirmed there had been CDI transmission, this could still be useful information to either encourage further audit or reassure staff that their existing practices were robust:

*But actually to be able to go to staff and to clinicians and say do you know what this VNTR [MLVA] typing is exactly the same as that. That means yeah that somehow yeah that patient is probably you know we’ve, we’ve um we’ve been in we’re implicated in that spread then if you like. Whereas if it’s a different typing then it just reinforces their practices or reassures them that their practices are robust* [93: MLVA Trust].

In some cases, typing results were useful in focusing infection control on specific clinical areas requiring further action. Ward antibiotic prescribing policies were mentioned as an example:

*I would say that the knowledge that the ribotyping has given us, has caused us to concentrate more on the antibiotic prescribing, type of thing. And so that’s one of the things we do now if we have say clusters of three, um is to go into that ward, look at antibiotics prescribing, do spot-checks on prescribing, communicate with clinicians about their prescribing, let them know what’s going on that sort of thing. So it’s enabled us to shift our focus really* [76: Ribotyping Trust].

*Well on [Ward name] we restricted, we changed the whole antibiotic policy for them. They had a totally different antibiotic policy to everybody else and on the back of that we changed our policy, our antibiotic policy, to have a separate section for the vulnerable elderly. […] So they don’t have a whole garden of antibiotics that they can choose from* [83: Ribotyping Trust].

However, typing results could also be viewed as unhelpful, especially if they failed to reinforce local good cleaning practice recommendations:

*And we spend a lot of time drilling people about the importance of commode cleaning so that’s something that’s happened over the last three or four years I suppose. And I suppose you could say that the ribotyping results didn’t really influence that because they would suggest that perhaps the commodes aren’t as important as we thought they were …[but] anybody’s entitled to a clean commode whether or not it’s going to give them an infection* [51: Ribotyping Trust].

##### Sub-theme: staff training

Table 3 shows that staff training was very rarely stopped as a consequence of a typing result. Additional staff training was reported at a similar frequency to audits of practice. In the latter case, interviews demonstrated that extra training was closely linked to audits, with the audit identifying an underlying practice issue which required targeted training:

*So, for instance, we have one outbreak with ribotype ‘001’ which was in fact confirmed by VNTR [MLVA] subsequently as all the same strains in ‘001’, and that was simply failure to isolate the patient index case in a timely manner. But that led to a whole load of specific actions and the training of the ward staff by the ward manager and so forth. So any defects in training, or additional training, tend to get picked up in the outbreaks* [94: Ribotyping Trust].

Well if the ribotyping has shown a confirmed outbreak then the root cause analysis - that’s undertaken. And that leads to an action plan. And one of the actions may be further education and staff training. [106: Ribotyping Trust].

#### Theme 4: indirect benefits

Although most typing results (55%) were reported not to have any direct impact on IC measures (see Table 3), at the same time, overall 88% of test-impact forms recorded that the recipient ‘agreed’ or ‘strongly agreed’ that the test had aided their management. The interviews therefore explored why a value was placed on typing results even though they had no direct impact on IC management. The explanations offered centred on more subtle organisational or indirect clinical benefits.

##### Sub-theme: organisational culture

When asked to explain, some interviewees described changes to organisational culture, which were real but difficult to quantify. It was explained by one interviewee that typing results had acted like a catalyst on the organisation:

*I just think, as I say I can’t tell you what difference it can make. I mean I’ve told you but it has really made a difference. When we started to use the VNTR [MLVA] typing it was a step change for us. And I am clear we wouldn’t be where we are without it……it has, it’s been like a catalyst and it enforces, reinforces good practice, and it exposes poor practice and that for me is you know it’s about what you actually do isn’t it on the ward* [39: MLVA Trust].

##### Sub-theme: reassurance/confidence building

Interviewees also thought that typing results had increased professionals’ confidence in their intuitive decision-making. This reassurance was especially important when dealing with vulnerable patients such as the elderly:

*Um I would think that, for me that would be, it’s the icing on the cake really. It’s that extra reassurance that yes they’re all the same sub-type as well .. yes, the care, the augmented care, because the elderly wards would be the ones where you’d really want to be sure that patients are, that patients are being managed appropriately* [84: Ribotyping Trust].

*But almost saying well this wasn’t you know a breach it probably was related to types of person coming in with it from a Nursing Home or whatever, and our control measures are still appropriate is so, so helpful* [55: Ribotyping Trust].

*Well I suppose just to reiterate just how useful and fascinating I’ve found it [i.e. typing], and I hope very much that it’s not on some funding or other that’s about to be withdrawn because it has certainly given us a great deal of confidence to say it’s unlikely that they’ll be the same.* [54: Ribotyping Trust].

##### Sub-theme: epidemiological value

The underlying clinical value most commonly mentioned in interviews was an improved understanding of the epidemiology of *C. difficile*. An understanding of the longer-term pattern of different strains on the wards or in the hospital more widely was very useful, especially for epidemic strains:

*Generally speaking, it’s given us an overview of the epidemiology… so essentially what it’s told us is that over time we’ve got rid of the three main epidemic strains, they have vanished* [1: Ribotyping Trust].

*We’re worried that .. people are dispersing all around you know like Trojan Horses round the trust and certainly we felt that this data…. will help support the fact that there is a nucleus where contamination occurs over a period of time and then people got shot off.* [20: MLVA Trust].

*So the patients may be linked when they’re not even on the same ward. But they both were on the same ward, previously […] let’s say if they’ve both been through Ward A but then one gets C. diff on Ward B, and one gets C. diff on Ward C. Previously, we wouldn’t have linked them at all. But now we are able to* [9: Ribotyping Trust].

*What would be useful is matching things not just to what we think is a problem on a ward because it can be wider, it is looking outside…because we’re blinkered. We just focus on that incident that we well know has got an [extended] incubation period and people who end up on the ward may have been somewhere else. I mean I just wondered if a centre had a year’s worth of data looking through every case whether you’d start seeing patterns* [23: MLVA Trust].

In this context, the more discriminatory power of MLVA typing results had a clear advantage. This information could be helpful in confirming or overturning assumptions based simply on CDI ribotyping patterns:

*So when we did have a few ‘027’s, we did a lot of work, and we thought we’d track them back and we could explain them. You know for example we said that this person was next to that person and they were in the medical assessment unit. And when we eventually got the sub-typing back it clearly wasn’t right at all. Our assumptions were wrong* [55: Ribotyping Trust].

One interviewee identified the potential for wider use of more discriminatory MLVA information to examine transmission routes not just in the hospital, but beyond, although this has not been undertaken:

*If we could actually map the types of the sub-types across the health-economy we might be able to track down well actually no it’s not on the ward, it’s .. it’s at the bingo down the road or, you know, this community centre, or whatever* [24: MLVA Trust].

## Discussion

Qualitative research evidence on the experiences of staff involved in trials of new diagnostic tests has been highlighted as important by bodies such as NICE [3], although it remains limited. As an example, evidence on the practical use of new typing tests for *C. difficile* in a real-world hospital context is lacking. Information from hospital staff who use these tests, particularly evidence on how typing information influences their infection control decisions during periods of increased CDI incidence and what broader benefits typing provides, remains a significant gap in the literature. As new rapid tests with increased discriminatory power to identify *C. difficile* strains are developed and promoted it is important to gather this evidence. The assumption is that providing such tests will lead to measurable benefits. However, the actual value of a new test is complex and will depend not only on what information it provides and how quickly, but also how this is combined with other organisational factors [2].

The findings reported here from a trial comparing a new, more rapid and discriminatory typing test (MLVA) with slower PCR-ribotyping help to address some of these questions. In our study, hospital staff highlighted three important changes in context which had influenced the use of CDI typing information at grass roots level. The first was a fall in the incidence of cases, with a 64% drop reported nationally between 2007 and 2010 [26], and linked to this the fact that typing information had shown that less than one in three PIIs involved infection transmission. Together this meant fewer instances in which decisions about implementing IC measures had to be made. In addition, the introduction of financial fines for confirmed CDI outbreaks in the UK had increased the perceived value of MLVA information for the organisation. An increased power to differentiated strains had reduced the number of outbreaks trusts had to report, and therefore reduced exposure to the risk of a financial penalties for the hospital [19].

In terms of direct clinical use of typing results, the picture emerging from our interviews is one of complex decision-making and limited flexibility to wait for test information when faced with a period of increased CDI incidence. The need for more rapid information is the most important factor limiting direct test utilisation. This was true both for existing ribotyping and for more rapid MLVA. When pressed, staff stated they might be prepared to wait 2-3 days before implementing IC measures. But, even the more rapid MLVA test could not provide results within this time frame. In the period while waiting for a typing result, IC measures were therefore still automatically triggered by hospital protocols. In some hospitals these protocols might now even be triggered by the first CDI case, before a second case was confirmed and therefore before typing was requested.

In terms of infection control measures, for CDI a growing evidence base has shown that a number of measures are effective. The primary measure shown to be effective in preventing transmission is rapid isolation (within two-hours). This is known to be the single most effective intervention to prevent transmission [11]. Interviewees reported that decisions on patient isolation were now easier to implement because of the lower numbers of CDI cases and reduced pressure on isolation facilities. The next most effective measure to prevent transmission is cleaning. After a few hours, airborne contamination will have occurred and surfaces will become ‘seeded’ with *C. difficile*. Following this, any area occupied by a CDI patient should be cleaned with various cleaning agents and germicides [27]. In some cases, more expensive dry-mist hydrogen peroxide decontamination of the whole area may be required to reduce the number of colony-forming units [28,29]. In interviews, initial decisions on cleaning were rarely linked directly to a typing result; hospital protocols were once again the main driver. But, additional ‘enhanced’ cleaning might be activated following typing information. Also, where existing cleanliness indicators were shown to have failed to provide early warning of a problem, a new scoring system might be introduced [30].

In addition to actions such as isolation and cleaning, regular audit of compliance with various infection control policies, including antimicrobial prescribing, are shown to have an effect on transmission [31]. In the present study, initial decisions on such audits were seldom influenced by typing; instead these were triggered by hospital protocols. Interestingly, audits of practice were the only IC measure where typing results were more likely to lead to an activity being stopped rather than initiated. In such cases, typing results might provide re-assurance that existing practices are robust and that further audits are unnecessary. Changes to ward policies (e.g. antibiotic prescribing policies) were also reported as a result of typing information. Extra staff training, initiated following a typing result, was usually linked to instances where additional audits had been undertaken. Test results could also occasionally be viewed as unhelpful in terms of improving staff practice if an audit identified that improvements were required but lab results subsequently indicated that no transmission had taken place. Expensive decisions on ward closures were only rarely influenced by a typing result. Typing information never stopped a planned closure but might lead to one being initiated, usually involving partial closure and cleaning of bays.

Any reduction in laboratory turnaround time for CDI typing tests should be set within the broader context of the overall hospital testing process. Although MLVA reduced typing turnaround time by over 8 days, there was no impact on the longer period (14 days) between identification of a PII and receipt of samples in the typing laboratory. Furthermore, the requirement to wait up to 28 days for a second CDI case to occur before a PII could be declared made the overall ‘testing period’ extremely protracted. Set against these timelines, a reduction of 8 days would have limited influence. At the same time, IC measures were increasingly being initiated during the 28 day period before a PII was declared and before the typing test was requested. The initial test used within a hospital to confirm infection is also important in the whole chain of testing and infection control. Currently, the optimum test for initially diagnosing *C. difficile* infection is being questioned in the UK because the assays commonly used in hospitals for toxin detection have been demonstrated to have poor sensitivity and specificity [32]. Combination testing has recently been advocated [33].

In the context describe above, the imperative to wait for a typing result before making a decision about whether or when to implement IC measures is reduced, tipping the balance towards immediate action. Interview evidence confirmed this, indicating that the main direct clinical benefits from typing results were refinements to IC measures already set in motion, or reassurance (after the event) that correct decisions had been made. This would appear to offer an explanation for the fact that the trial could demonstrate no significant impact on the number of CDI cases in hospitals with access to more rapid MLVA typing tests. At the same time, MLVA typing results were four times more likely than ribotyping to be rated ‘strongly agree’ in terms of their perceived value in aiding management. However, interviews also indicated that MLVA results could be complex to understand, not always providing as clear cut an answer as ribotyping. This meant that consultant microbiologists were not able to delegate this task to more junior staff and might themselves even need further advice from the typing laboratory. In some cases hospital staff reported that, because of a lack of certainty in the new technology, MLVA results which indicated no transmission might not be believed, perhaps linked to worries over correct sampling. Finally, the added organisational and management effort associated with requesting a typing test (identifying cases occurring within 28 days of each other and arranging for samples to be extracted and sent to the regional laboratory) could also act as a barrier. In one trust, difficulties in setting up an efficient system for ordering MLVA typing tests had led to underutilisation of the new service.

Overall, the interview data did not demonstrate any substantive differences between the MLVA and ribotyping group in direct use of typing results. At the same time, when asked to describe the value of typing information the answer most commonly given was an improved understanding of the underlying epidemiology of CDI strains in the hospital. This benefit is not, of course, dependent on the speed of the test result and enhanced by the ability of MLVA to discriminate between various sub-types. When asked to compare the more discriminatory information provided by MLVA with ribotyping this was, as might be expected, thought to be of most value in the context of common ribotypes where the added information provided could help confirm whether transmission had actually occurred. In particular, respondents identified the potential for more discriminatory MLVA information to be useful when considering patient movements between wards or beyond the hospital.

Following the introduction of an NHS Ribotyping Network in 2007, *C. difficile* figures in English hospitals have declined markedly and deaths associated with CDI have also decreased. It has been suggested that access to a ribotyping service may have facilitated better local control of CDI cases, leading to this decrease [34]. However, a 2009 report by the National Audit Office (NAO) on Healthcare Associated Infections in Hospitals in England found that, although twenty nine per cent of hospital trusts had managed to reduce CDI by over 50 per cent, 19 per cent of trusts were found to have had an increase [35]. The NAO reported that leadership, performance management and clinical practice were important factors in differentiating trusts.

The methodological challenges associated with performance of qualitative studies alongside randomised controlled trials, and the associated benefits, are well documented for complex healthcare interventions, but not for diagnostic tests [36,37]. In diagnostics, the focus still remains largely on technical evaluation of test performance. The process of test utilisation and the experiences of staff using the tests are largely ignored. This is not surprising since in diagnostics even randomised controlled trials evaluating the impact of tests on patient outcomes are rare [38]. As a result, considerable methodological challenges remain. Even though guidelines are being developed for broader assessment of the value of diagnostic studies, these do not yet consider the role of qualitative research [39]. The main limitation of the present study is that, although in-depth qualitative data was collected, this was necessarily dependent on the views of selected senior staff in a relatively small sample of hospital trusts. Even so, the main evidence and themes emerging as described above are consistent.

The evidence from our study highlights the real-world issues that hospitals face in trying to integrate typing test information into their management of possible CDI outbreaks. Improved control of CDI transmission remains an economic as well as a clinical imperative. The cost of *C. difficile* infection is considerable; it has been estimated as €3 billion/year in the EU [8], with increased hospital length of stay plus subsequent re-admissions to hospital as the main cost drivers [40]. The British Medical Association has concluded that reductions in hospital-acquired infections such as CDI can only be achieved through strong organisational commitment and implementation of policies that are practicable and effective in prevention and control [31]. This study shows that strong hospital protocols, used to direct infection control procedures, are in use in NHS hospitals. However, more research is needed to explore how best to provide a typing service. Particularly relevant is a need for agreement between hospital trusts and regional laboratory on accessing the service, presentation of results, and optimum use of typing information.

## Conclusions

Although the majority of typing results were considered to be of value, typing information was reported to have limited direct impact on infection control measures during a PII. The timing of typing results appeared to be a major limiting factor. Even with a reduction of 8 days between the delivery of MLVA and ribotyping results, the turnaround time for both was considered too long. Implementation of IC measures was driven by hospital protocols and commenced prior to receiving the typing result. The MLVA typing service was valued predominantly for the more discriminatory typing information provided, rather than its increased speed. This provided benefits for professionals either in terms of reassurance about decisions already taken or by allowing fine tuning of infection control measures. The main reason given for valuing MLVA information was an improved understanding of the epidemiology of CDI strains in the hospital. There remains the potential for wider use of typing information to examine transmission routes. Impact on organisational culture was more subtle, and difficult to quantify.

## Abbreviations

CDI: *Clostridium difficile* infection; H2O2: Hydrogen peroxide; HAIs: Hospital acquired infections; HPA: Health Protection Agency; IC: Infection control; NAO: National Audit Office; NHS: National Health Service; PCT: Primary care trust; SHA: Strategic health authority; UK: United Kingdom.

## Competing interests

The authors declare that they have no competing interests.

## Authors’ contributions

AS, KH and PMH conceived the idea for the study. AS undertook the interviews. The manuscript was reviewed and approved by all authors. All authors had full access to data in the study.

## Pre-publication history

The pre-publication history for this paper can be accessed here:

http://www.biomedcentral.com/1471-2334/14/154/prepub

## Supplementary Material

Additional file 1 Test-impact form. Click here for file
