# Supplementary material for: How do hospital professionals involved in a randomised controlled trial perceive the value of genotyping vs. PCR-ribotyping for control of hospital acquired C. difficile infections?
Source: BMC Infect Dis. 2014 Mar 21;14:154. doi: 10.1186/1471-2334-14-154 (PMC3997920; doi:10.1186/1471-2334-14-154)
Supplement: Additional file 1 — Test-impact form. [file 1471-2334-14-154-S1.docx]

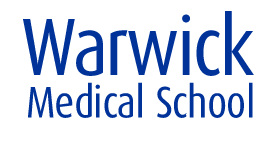
**ADDITIONAL FILE 1: TEST-IMPACT FORM**

**Control of hospital acquired *Clostridium difficile* infection (CDI)**

**by typing information**

**DATA COLLECTION FORM following CDI typing result**

**Lab cluster number: Date result sent:**

**Trust:**  **Ward:**

**Outbreak confirmed: Yes/No**

*You were recently sent the typing result above. As part of the study we would like to know if any actions were introduced or stopped as a result of the typing results.*

**Q1.** After receiving this result were any additional actions taken?

| Yes |  | *If Yes please go to* Q2 *→* | No |  | *If No please continue to* Q3 *→* |
| --- | --- | --- | --- | --- | --- |

**Q2.** If Yes, which of the following additional actions were taken? *(indicate all that apply)*

Yes

| Ward closure |  | *For how many days?* | Days |
| --- | --- | --- | --- |
|  |  |  |  |
| Increase in frequency of cleaning |  | *For how many days?* | Days |
|  |  |  |  |
| Change in cleaning product used |  | *For how many days?* | Days |
|  |  |  |  |
| Use of steam cleaning |  |  |  |
|  |  |  |  |
| Use of hydrogen peroxide vapour |  |  |  |
|  |  |  |  |
| Audits of practice |  |  |  |
|  |  |  |  |
| Teaching/training of staff |  |  |  |

| OTHER |  |  | *Brief details please:* |
| --- | --- | --- | --- |
|  |  |  |  |

**Q3.** After receiving this result were any measures that had been implemented prior to the result stopped?

| Yes |  | *If Yes please go to* Q4 *→* | No |  | *If No please continue to* Q5 *→* |
| --- | --- | --- | --- | --- | --- |

**Q4.** If Yes, which of the following actions were stopped? *(indicate all that apply)*

Yes

| Ward closure |  |  |
| --- | --- | --- |
|  |  |  |
| Increase in frequency of cleaning |  |  |
|  |  |  |
| Change in cleaning product used |  |  |
|  |  |  |
| Use of steam cleaning |  |  |
|  |  |  |
| Use of hydrogen peroxide vapour |  |  |
|  |  |  |
| Audits of practice |  |  |
|  |  |  |
| Teaching/training of staff |  |  |

| OTHER |  |  | *Brief details please:* |
| --- | --- | --- | --- |
|  |  |  |  |

**Q5.** Do you consider that this typing result has aided your management?

|  | Strongly agree |  | Agree |  | Disagree |  | Strongly disagree | *(Please mark* ***one*** *box only)* |
| --- | --- | --- | --- | --- | --- | --- | --- | --- |
